# Evaluating the Effectiveness of mHealth Application Interventions to Promote Breastfeeding Amongst Pregnant Women and Postpartum Mothers: A Global Systematic Review and Meta‐Analysis

**DOI:** 10.1111/mcn.70230

**Published:** 2026-08-17

**Authors:** Yim Ching Yau, Mary Fewtrell, Martin Ming Him Wong, Ping Him Lam, Kerrie Stevenson

**Affiliations:** ^1^ Institute of Epidemiology and Health Care University College London London UK; ^2^ Great Ormond Street Institute of Child Health University College London London UK; ^3^ Department of Computing The Hong Kong Polytechnic University Hong Kong SAR China; ^4^ Faculty of Population Health Sciences University College London London UK

**Keywords:** breastfeeding, breastfeeding support, exclusive breastfeeding, lactation support, mHealth, mobile health, mobile health applications

## Abstract

Despite global recommendations, less than 40% of infants ≤ 6 months are exclusively breastfed (EBF) reflecting socio‐economic, cultural, and systemic barriers. Mobile health (mHealth) interventions (delivered through mobile phone applications to support medical/public health practices) have been developed to support breastfeeding, but their effectiveness remains unclear. In this systematic review we searched PubMed, Embase, CINAHL, Web of Science, and Cochrane Library from 1 January 2010 to 1 May 2025 for randomised controlled trials in English involving pregnant or postpartum women up to 1 year, assessing the effectiveness of any form of mHealth intervention aimed at the mother, excluding qualitative studies (PROSPERO: CRD420251032811). The main outcome was EBF ≤ 6 months; Secondary outcomes were EBF at later time points, overall breastfeeding duration and breastfeeding self‐efficacy up to 12 months. Quantitative synthesis used Harvest plots, binomial calculations, and meta‐analysis. Of 3102 retrieved records, 13 studies (3269 women) were included. Interventions typically combined mobile apps with text messaging or telelactation. Binomial calculations/harvest plots suggested strong evidence for improved breastfeeding self‐efficacy, knowledge, and confidence, with less evidence for effects on initiation, exclusivity, and duration. Meta‐analysis suggested increased breastfeeding self‐efficacy (Hedges′ *g* = 1.08, 95% CI 0.07–2.1, *p* = 0.04) but with high heterogeneity (*I* ^2^ = 90.7%), reflecting diverse populations, intervention types, and contexts. Engagement was affected by maternal, socio‐cultural, and structural factors, underscoring the need for tailored, accessible, context‐sensitive interventions. mHealth interventions may improve breastfeeding self‐efficacy, knowledge and confidence, particularly when incorporating interactive, personalised features such as real‐time feedback or telelactation.

## Summary

- Mobile application–based interventions significantly may enhance maternal breastfeeding self‐efficacy and knowledge among pregnant and postpartum women.
- Although there are some improvements in exclusive breastfeeding rates, initiation, and duration were observed, the effects remain inconsistent across studies and follow‐up periods.
- Interventions incorporating interactive and personalised features, such as real‐time feedback or telelactation support, demonstrated greater effectiveness than static educational applications.
- Digital literacy, access inequality, and cultural relevance emerged as key barriers influencing engagement and sustained use of mHealth breastfeeding interventions.
- Further high‐quality and culturally responsive randomised controlled trials are warranted to establish long‐term effectiveness and implementation feasibility across diverse populations.

## 1 Introduction

Breastfeeding is widely recognised as a critical public health intervention that enhances infant and maternal health, reducing infant mortality, improving cognitive development and protecting against illnesses such as diarrhoea and pneumonia, while lowering maternal risks of breast and ovarian cancers, aiding postpartum recovery and supporting birth spacing (Rollins et al. 2016; Victora et al. 2016; World Health Organisation 2024). The World Health Organization (WHO) and UNICEF recommend exclusive breastfeeding (EBF) for the first six months of life, followed by continued breastfeeding alongside complementary foods for up to two years or more (UNICEF 2023; World Health Organisation 2017, 2024). Despite these well‐established benefits, globally, less than 40% of infants under 6 months are exclusively breastfed, far below the 70% global target for 2030 (Gertosio et al. 2016). Barriers to breastfeeding are multifaceted, including socio‐economic, cultural, and systemic factors such as limited skilled support, misinformation, stigma, early return to work, and unsupportive environments (Dowling et al. 2012; Dowling et al. 2018; Kavle et al. 2017; McFadden et al. 2017).

Mobile health (mHealth) technologies have emerged as innovative strategies to address these challenges by leveraging mobile phones and wireless platforms to support health behaviours (World Health Organisation 2011). mHealth applications (apps) deliver interactive, personalised, and multimedia content that incorporates behaviour change techniques such as goal setting, self‐monitoring, and reminders to promote sustained engagement (McKay et al. 2018; Ziebart et al. 2024). Their scalability, cost‐effectiveness, and ability to integrate education, tracking, and professional or peer support make them valuable for timely, accessible breastfeeding support, even in underserved regions (Lee et al. 2015; Sondaal et al. 2016).

Existing research remains inconsistent across contexts due to methodological heterogeneity, limited sample sizes, and varied outcomes (Chen et al. 2018; Daly et al. 2018; Ishaque et al. 2025; Sondaal et al. 2016; Ziebart et al. 2024). Also, evidence on long‐term outcomes, maternal morbidity, and the inclusion of vulnerable populations remains scarce. There is evidence supporting mHealth's effectiveness in promoting breastfeeding (DeNicola et al. 2020; Sondaal et al. 2016). DeNicola et al. (2020) concluded that it could improve obstetric outcomes and breastfeeding. Sondaal et al. (2016) reported that mHealth interventions in low‐and‐middle‐income countries (LMICs) improved maternal and neonatal service utilisation, doubling antenatal care attendance (OR 2.39, 95% CI 1.03–5.55) and reducing perinatal mortality by 50% (OR 0.50, 95% CI 0.27–0.90). App‐based interventions have improved short‐term breastfeeding initiation and exclusivity (McKay et al. 2018; Ziebart et al. 2024), but cultural adaptability and sustained engagement in diverse settings such as Brazil and Southeast Asia continue to present challenges (Diniz et al. 2019; Leal et al. 2023; Te Ku Nor and Wee 2023). Persistent barriers such as limited smartphone access, poor internet connectivity, and low digital literacy restrict participation in low‐income and rural settings (Diniz et al. 2019; Knop et al. 2024; Qian et al. 2021), while inconsistent app content, lack of interactivity, and poor usability further constrain effectiveness (McKay et al. 2018; Daly et al. 2018; Mieso et al. 2022; Ziebart et al. 2024). Systematic reviews also reinforce these limitations, reporting small, heterogeneous samples that prevented firm conclusions on maternal knowledge, behaviour change, or perinatal outcomes (Daly et al. 2018), significant improvements in exclusive breastfeeding rates, self‐efficacy, and attitudes, but no effect on initiation within an hour of birth and limited evidence on long‐term breastfeeding (Qian et al. 2021), demonstrated benefits of smartphone‐based education and counselling across multiple countries but noted substantial heterogeneity and variable adherence (Pratiwi et al. 2023), and, inconsistent app quality and evaluation standards (Ziebart et al. 2024). Collectively, these reviews reveal gaps in sample size, methodological consistency, outcome measurement, and population representation, underscoring the need for rigorous, standardised, and culturally tailored mHealth interventions to support sustained breastfeeding outcomes.

To address these gaps, this systematic review aims to evaluate mobile application–based interventions designed to promote breastfeeding worldwide by identifying and categorising intervention types across regions, assessing their effectiveness in improving breastfeeding outcomes such as initiation, exclusivity, and duration, and exploring the facilitators and barriers influencing their success in diverse socio‐cultural and economic contexts. The Capability, Opportunity, Motivation, and Behaviour (COM‐B) model (Michie et al. 2011) is applied to analyse individual studies and synthesise findings by region. Table 1 presents the definitions used throughout this paper.

**Table 1:** Definitions of terminologies.

Terminologies	Definitions
mHealth	Use of mobile devices and wireless technology to support medical and public health practices (World Health Organisation 2011).
Mobile application‐based interventions	Interventions or programs delivered through mobile phone applications to support medical and public health practices.
Exclusive breastfeeding (EBF)	Feeding only breast milk (no food or drink) (WHO, 2023). (The study will also adopt the definitions of EBF as used in each included study to ensure comparability and inclusivity of available evidence.)
Breastfeeding Self‐efficacy	A mother's confidence in her ability to breastfeed her new infant (Dennis 1999)^a^.
Effectiveness	The extent to which an intervention achieves its intended outcomes in real‐world settings (Rychetnik et al. 2002).
Gross National Income (GNI)	A total amount of factor incomes earned by the residents of a country. (The World Bank 2024).
Upper‐middle or High‐Income Countries (UMICs and HICs)	Countries with a Gross National Income (GNI) per capita greater or between $4495 and $13,935 (The World Bank 2024).
Low‐ and Middle‐Income Countries (LMICs)	Countries with a Gross National Income (GNI) per capita lower or between $1136 and $4495 (The World Bank 2024).
Telelactation	Services that connect breastfeeding mothers to remotely located healthcare professionals using any real‐time audio‐visual technology (Kapinos et al. 2019).

^a^ Breastfeeding self‐efficacy was defined, consistent with Dennis' conceptualisation, as a mother's confidence in her ability to successfully breastfeed her infant, regardless of whether this is her first or a subsequent child.

## 2 Methods

This systematic review was conducted in accordance with Preferred Reporting Items for Systematic reviews and Meta‐Analyses (PRISMA 2025) guidelines. We searched PubMed/MEDLINE, Embase, CINAHL, Web of Science, and the Cochrane Library for studies published in English between 1 January 2010 and 1 May 2025, using terms related to breastfeeding, mHealth/mobile interventions, and study design. Eligible studies were randomised controlled trials of mHealth interventions for pregnant women or postpartum mothers intending to breastfeed, reporting at least one breastfeeding‐related outcome. Two reviewers independently screened records, extracted data, and assessed risk of bias using RoB 2 (Cochrane Methods Bias 2025; Higgins et al. 2019a), with disagreements resolved by discussion or third‐reviewer adjudication. CONSORT checklists were used for quality assessment (Cuschieri 2019). Narrative synthesis was conducted following Cochrane guidance, and meta‐analysis was performed where outcomes were sufficiently comparable; heterogeneity was assessed using *I* ^2^. Details of methods, search strategy and search terms are provided in Supporting Information S1: Materials [Link], [Link] and 3.

**Figure 1:**
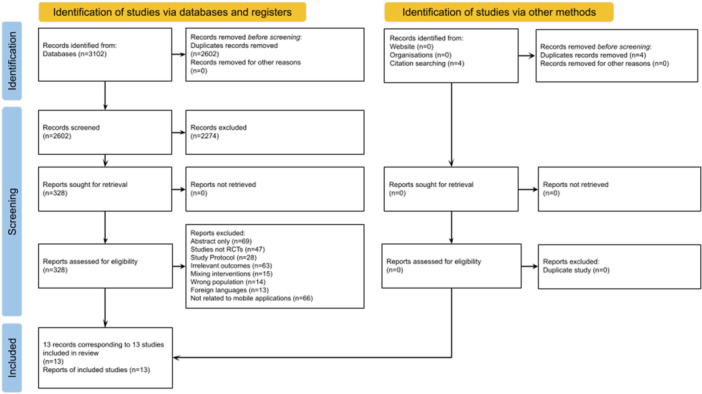
Preferred reporting items for systematic reviews and meta‐analysis (PRISMA) flow diagram.

Inclusion and exclusion criteria were pre‐defined with input from experts by experience, the supervisor, and the relevant literature (Table 2). A flowchart summarises the selection process (Figure 1). Data synthesis is summarised in Supporting Information S1: Material 1.

**Table 2:** Inclusion and exclusion criteria.

	Inclusion criteria	Exclusion criteria
Population	Pregnant women or postpartum mothers up to 12 months postpartum intending to breastfeed	Health professionals Family members
Intervention	mHealth strategies via apps, including educational content, peer support, reminders (SMS/text), or telehealth consultations with lactation consultants	Interventions unrelated to breastfeeding Indirect breastfeeding interventions (e.g., healthcare worker training) Purely face‐to‐face Hybrid approaches without/little mobile application components
Study Design	Randomised controlled trials (RCTs)	Observational, non‐randomised, qualitative, or mixed‐methods studies
Outcomes	At least one breastfeeding‐related measure (or other specify):	No breastfeeding‐related outcomes reported
	Initiation Exclusive breastfeeding Duration Maternal knowledge Attitudes Self‐efficacy Engagement with the mHealth tool	
Publication	Published in English Peer‐reviewed literature Full text available Published between January 1, 2010, and May 1, 2025	Not in English Full text unavailable/No access

*Note:* Mixing interventions refers to those that are not primarily mobile‐based, and involves face‐to‐face interventions.

### 2.1 Ethical Statement

Exempt from full ethical review, the project upheld ethical standards through transparent methodology, proper citation, and honest reporting, with reliability ensured via duplicate screening, data extraction, bias assessment, PRISMA adherence, and PROSPERO registration (reference: CRD420251032811).

## 3 Results

The search retrieved 3102 records, of which 328 full texts were reviewed after initial screening. 13 studies were included, comprising data from 3269 pregnant women and postpartum mothers. A total of 13 RCTs were included, with five studies (38.5%) conducted in low‐and middle‐income countries (LMICs). Studies were published between 2019 and 2025, with 12 (92.3%) since 2020. Intervention characteristics are summarised in Table 3, with full details in Supporting Information S1: Material 4. Interventions were mHealth application‐based and classified as: (1) self‐guided apps providing educational content and breastfeeding resources (38.5%, *n* = 5), (2) apps combined with live support, such as telelactation or expert consultations (38.5%, *n* = 5), and (3) blended interventions integrating apps with additional telesupport via SMS or phone calls (23%, *n* = 3).

**Table 3:** Characteristics and references of included studies.

Characteristic	*n* (%)
Study design:	
Randomised control trials (RCTs)	13 (100.0%)
Pilot study	3 (23.1%)
Country:	
United States	5 (38.4%)
Iran	3 (23.1%)
Turkey	2 (15.4%)
Nigeria	1 (7.7%)
Spain	1 (7.7%)
Uganda	1 (7.7%)
UMICs and HICs rate (Total)^a^	8 (61.5%)
LMICs rate (Total)^a^	5 (38.5%)
Publication year:	
2020–2025	12 (92.3%)
< 2020 (i.e., 2019)	1 (7.7%)
Perinatal period:	
Postpartum	8 (61.5%)
Perinatal period	5 (38.5%)
Intervention categories:	
Self‐guided mobile applications providing educational content and breastfeeding resources:	5 (38.5%)
Mobile applications combined with live support	5 (38.5%)
Blended interventions involving mobile apps with additional telesupport through SMS/telephone calls	3 (23.0%)
Intervention contents^b^	
Online educational materials (including videos and audios)	10 (76.9%)
Direct access/video calls to support (e.g., obstetrician/nurses)	6 (46.2%)
Appointment reminders/notifications	5 (38.5%)
Frequently asked questions and corresponding answers	4 (30.8%)
Multilingual support (Two languages or more)	3 (23.1%)
Resources accessible at the institution	2 (15.4%)
Strategies for working mothers	2 (15.4%)
Timelines for infant needs	2 (15.4%)
Maternal products recommendations for breastfeeding	1 (7.7%)
Main outcomes:	
Exclusive breastfeeding (EBF) rate^c^ (total studies)	6 (46.2%)
At postpartum Day 2	1 (7.7%)
At 4–6 weeks postpartum	3 (23.1%)
At 3 months postpartum	1 (7.7%)
At 6 months postpartum	3 (23.1%)
Breastfeeding self‐efficacy (total studies)	6 (46.2%)
At 1st day and 3rd day and the 1/2/3/4/5/6/7th week postpartum	1 (7.7%)
At 4–6 weeks postpartum	2 (15.4%)
At 7–8 weeks postpartum	2 (15.4%)
At 3 months postpartum	1 (7.7%)
At 6 months postpartum	1 (7.7%)
At 12 months postpartum	1 (7.7%)
Other relevant outcomes:	
Breastfeeding duration and frequency	5 (38.5%)
Breastfeeding initiation	3 (23.1%)
Knowledge (maternal/breastfeeding‐related)	3 (23.1%)
Breastfeeding attitude/satisfaction	2 (15.4%)
Breastfeeding‐related behavioural changes/practices	2 (15.4%)
Breastfeeding effectiveness, reduced difficulties/challenges	1 (7.7%)

^a^ HICs: High‐income countries; LMICs, low‐and‐middle‐income countries; UMICs, upper‐middle income countries.

^b^ Percentages do not sum to 100% as individual studies often incorporate multiple intervention components.

^c^ EBF rates are reported with specified period only, not including those without clearly mentioned period or mixed within any form of BF, that is, mixed feeding.

The studies included diverse pregnant and postpartum women, mainly first‐time mothers. Most (61.5%, *n* = 8) focused on primiparous women in the third trimester intending to breastfeed, while 30.8% (*n* = 4) (Acar and Şahin 2024; Uscher‐Pines et al. 2025; Vila‐Candel et al. 2024; Karaçay Yıkar and Nazik 2024) involved postpartum mothers with infants aged birth to 2 years. Two studies (15.4%, *n* = 2) (Lewkowitz et al. 2020; Sosanya et al. 2025) targeted young mothers (14–19 years) or those with limited education in LMICs settings. Participants were generally healthy, aged 14–35 years, with follow‐up from postpartum Day 2 to 1 year. For further details please refer to Supporting Information S1: Material 5.

### 3.1 Study Characteristics

Five (38.5%) interventions began during pregnancy, and eight (61.5%) were initiated postpartum, with durations ranging from 6 weeks to 12 months. Several studies included app usage tracking, personalised content, and breastfeeding monitoring tools, with varying levels of interactivity and healthcare provider involvement. Following Cochrane guidelines (Higgins et al. 2019b), all reported outcomes were categorised, identifying two primary outcomes: EBF and self‐efficacy, each reported in six studies (46.2%). Secondary outcomes included breastfeeding duration and frequency (5 studies, 38.5%), maternal knowledge and initiation (3 studies, 23.1%), attitudes or satisfaction, and behavioural changes (2 studies, 15.4%), and breastfeeding effectiveness, including reduced difficulties (1 study, 7.7%). Additional common core features included online educational materials (10 studies, 76.9%), direct access or video calls to healthcare personnel (6 studies, 46.2%), appointment reminders (5 studies, 38.5%), and frequently asked questions (FAQs) (4 studies, 30.8%). Other features addressed maternal needs, including strategies for working mothers (2 studies, 15.4%), infant care timelines (2 studies, 15.4%), institutional resources (2 studies, 15.4%), multilingual support (3 studies, 23.1%), and maternal product recommendations (1 study, 7.7%). All the interventions are free to mothers.

### 3.2 Risk of Bias Assessment

The risk of bias was assessed using the Cochrane Risk of Bias 2.0 (RoB 2) tool (Q1‐5) (Cochrane Methods Bias 2025), and the results are summarised in Supporting Information S1: Material 6. Of the 13 studies included, five studies (38.5%) did not provide sufficient information regarding the generation or concealment of the random allocation sequence, and similar concerns were noted in the reporting of blinding procedures for participants and personnel. While most studies clearly described outcome measurement methods, some lacked detail on whether outcome assessors were blinded. All studies reported the prespecified outcomes as outlined in their methods sections, and no study showed evidence of selective reporting. Overall, eight (61.5%) studies were judged to have a low risk of bias, while five (38.5%) were assessed as having some concerns. All RCTs adhered to CONSORT reporting standards (Cuschieri 2019).

### 3.3 Narrative Synthesis

#### 3.3.1 Participant Demographics and Contextual Characteristics

Maternal profiles varied significantly by regional economic status, particularly regarding breastfeeding experience. While participants were aged 14 to mid‐30s across all studies, a distinct divide in parity was observed: prior breastfeeding was common in UMICs and HICs (56%–75%) (de Mello Sa et al. 2025; Uscher‐Pines et al. 2019), whereas LMIC studies primarily featured primiparous mothers (Musiimenta et al. 2022; Sosanya et al. 2025). Across these contexts, age and parity were consistently identified as factors that modified both mHealth engagement levels and clinical breastfeeding outcomes.

#### 3.3.2 Impact of Interactive Support and Personalisation on Self‐Efficacy and Practice

The effectiveness of the interventions was divergent based on the level of human interaction provided. Interventions incorporating interactive components or personalised support, such as telephone follow‐ups (Abadi et al. 2024), telelactation consultations (Uscher‐Pines et al. 2025), and e‐consultancy (Karaçay Yıkar and Nazik 2024), reported the most consistent improvements in maternal self‐efficacy. In contrast, outcomes requiring long‐term behavioural change, such as sustained exclusive breastfeeding rates, were less consistently improved (Lewkowitz et al. 2020; Hongo et al. 2020). These findings highlight a trend where digital interventions effectively bolster psychological readiness but vary in their impact on sustained practice depending on design intensity.

#### 3.3.3 Socioeconomic and Educational Factors

Socioeconomic status and literacy levels functioned as one of the determinants of intervention accessibility. In UMICs and HICs, high school completion was standard and college education was prevalent (25%–88%) (de Mello Sa et al. 2025; Uscher‐Pines et al. 2019). Conversely, LMIC participants reported predominantly primary‐level education (93%–98%) and higher rates of informal or unpaid work (73%–75%) (Musiimenta et al. 2022; Sosanya et al. 2025). These disparities coincided with reports of limited app engagement in low‐income groups, where low literacy and financial constraints restricted the use of complex mHealth tools.

#### 3.3.4 Cultural, Social and Family Influences

The social environment served as a consistent facilitator of mHealth engagement, though the ethnic composition of study populations varied by region. In UMICs and HICs, study samples were primarily diverse, including Caucasian and Black participants (de Mello Sa et al. 2025; Lewkowitz et al. 2020), whereas LMIC samples were characterised by ethnic homogeneity (Musiimenta et al. 2022; Sosanya et al. 2025). Despite these demographic differences, marital and partner support was a universal facilitator, with high prevalence across all regions (53%–95%) correlating with improved breastfeeding and mHealth adherence (de Mello Sa et al. 2025; Musiimenta et al. 2022; Uscher‐Pines et al. 2019). Furthermore, extended family support, specifically from mothers and mothers‐in‐law emerged as a cross‐regional factor that enhanced intervention engagement and reinforced the educational content delivered via the mHealth platforms (Karaçay Yıkar and Nazik 2024).

#### 3.3.5 Maternal, Obstetric and Structural Factors

**Figure 2:**
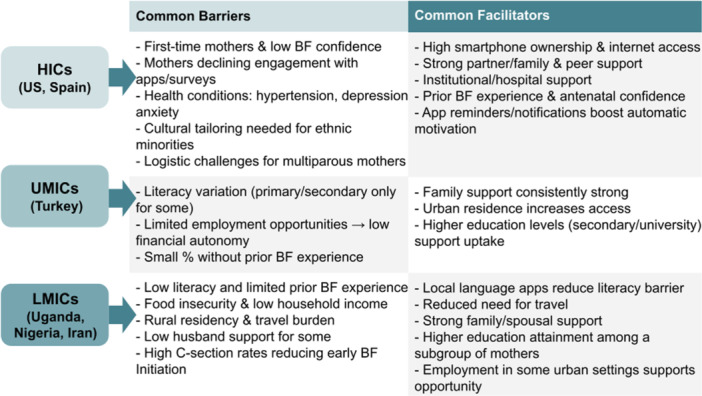
Summary matrix of barriers & facilitators across regions.

Structural barriers to mHealth adherence included obstetric history and technological infrastructure. While pregnancy planning was high globally (83%–95%), Caesarean section rates reached as high as 75% in some LMIC samples (Musiimenta et al. 2022; Seddighi et al. 2022). Despite these high rates, data regarding specific mHealth support for post‐C‐section breastfeeding remains limited. Furthermore, a ‘digital divide’ was evident in smartphone ownership, which was near‐universal in HICs (89%–97%) but restricted in LMICs by geographic distance from health facilities (8–20 km) and the associated costs of data and device maintenance (Musiimenta et al. 2022; Sosanya et al. 2025). Figure 2 summarises cross‐regional facilitators and barriers across HICs, UMICs and LMICs, with detailed breakdowns in Supporting Information S1: Material 7.

### 3.4 Harvest Plot Analysis

**Figure 3:**
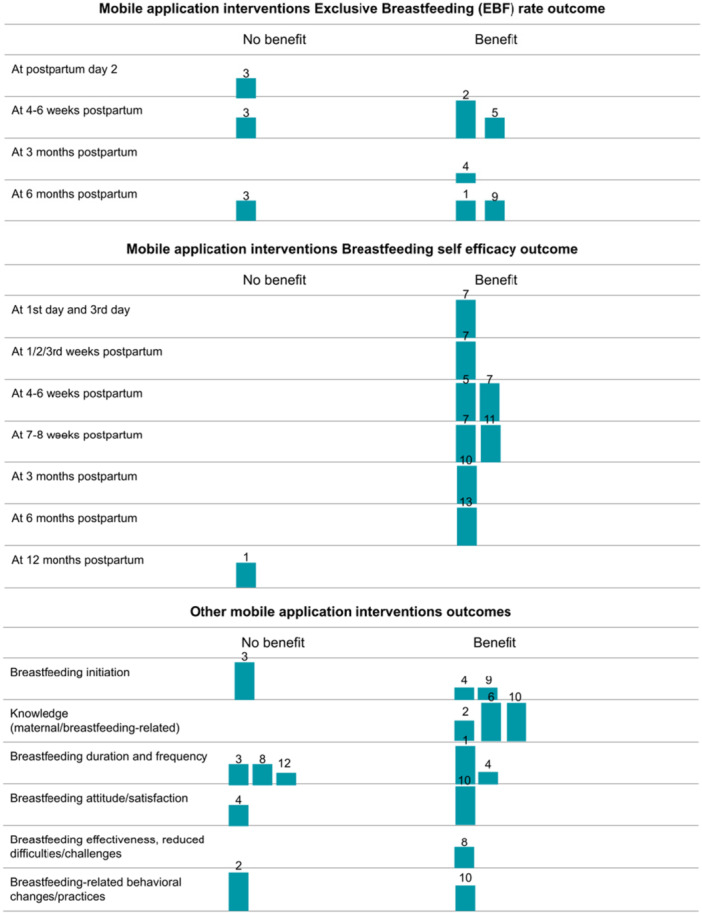
Harvest plots: Evidence for effectiveness of mobile application interventions. Evidence for effectiveness of the interventions included in this study, by main outcome category. This harvest plot is a ‘supermatrix’ presenting all included interventions and outcomes. Each bar represents a single study and is annotated with its reference number, corresponding to the following studies (First author year): 1. de Mello Sa et al. 2025; 2. Musiimenta et al. 2022; 3. Lewkowitz et al. 2020; 4. Uscher‐Pines et al. 2019; 5. Saucedo Baza et al. 2023; 6. Sosanya et al. 2025; 7. Karaçay Yıkar and Nazik 2024; 8. Acar and Şahin 2024; 9. Uscher‐Pines et al. 2025; 10. Seyyedi et al. 2021; 11. Seddighi et al. 2022; 12. Vila‐Candel et al. 2024; 13. Abadi et al. 2024. Bar height indicates study quality: strong (tallest), moderate (medium), weak (shortest).

Mobile application‐based interventions generally improved maternal breastfeeding outcomes, particularly self‐efficacy and knowledge, while effects on EBF, initiation, duration, and behavioural practices were more variable (Figure 3).

#### 3.4.1 EBF Across Postpartum Stages

All studies were prospective in design and collect data via surveys or questionnaires, Vila‐Candel et al. (2024) also include routine visits. EBF effects varied by postpartum period, with early gains (≤ 6 weeks) in 2/3 studies but mixed results at 3–6 months across six RCTs (Figure 3). For early outcomes, Lewkowitz et al. (2020) reported no day 2 benefit (36.6% vs. 35.7%; RR 1.02, *p* = 0.90). At 4–6 weeks, Musiimenta et al. (2022) achieved 100% EBF in the intervention group versus 81% in control, Saucedo Baza et al. (2023) reported 65% vs. 32% (*p* = 0.09), though Lewkowitz showed no sustained effect.

As for later outcomes, Uscher‐Pines et al. (2019) found a modest, non‐significant improvement (51.0% among intervention group vs. 46.0% among controls; *p* = 0.47). At 6 months, de Mello Sa et al. (2025) reported higher EBF in the intervention group (81.3% among intervention group vs. 60.0% among controls; *p* = 0.28). Uscher‐Pines et al. (2025) reported small Intent‐to‐treat (ITT) improvements (46.9% among intervention group vs. 44.1% among controls; *p* = 0.28), with significant gains among Black participants (42.7% among intervention group vs. 33.9% among controls; *p* = 0.02) and stronger effects in instrumental variable (IV) analysis, which uses randomisation as an instrument to estimate effects among actual adherers (8.4 percentage points, 95% CI 0.5–15.7; *p* = 0.03). This pattern of stronger subgroup effects amid modest overall ITT gains reflects common adherence challenges in telelactation studies. Sensitivity analyses suggested even larger improvements (11.6 percentage points; 95% CI 1.9–21.4; *p* = 0.02). This time‑varying, subgroup‑specific pattern suggests mHealth supports early EBF but sustained population effects require better adherence strategies and structural support.

#### 3.4.2 Maternal Confidence, Behavioral Change and Knowledge Gains

Self‐efficacy improved consistently across six RCTs (100% positive direction, Harvest Plot), strongest early postpartum and with interactive support, though effects attenuated by 12 months. This was most commonly assessed using the Breastfeeding Self‐Efficacy Scale (BSES) (Amini et al. 2019). Karaçay Yıkar and Nazik (2024) reported higher BSES scores from 2 to 7 weeks postpartum (*p* < 0.001), Seddighi et al. (2022) observed increases in the intervention group (48.26 ± 6.49 to 53.78 ± 12.61) versus declines in controls (49.11 ± 7.36 to 41.90 ± 17.98; *p* < 0.001), and Seyyedi et al. (2021) noted +26.85 ± 7.13 vs. +0.40 ± 5.17 (*p* < 0.001). Improvements persisted to 6 months (+22.05 ± 5.62 vs. +3.5 ± 8.6; *p* < 0.05 (Seddighi et al. 2022); 7.6 ± 7.8 vs. 1.2 ± 3.7; *p* = 0.001) (Saucedo Baza et al. 2023), but benefits were absent at 12 months (median = 52 vs. 55; *p* = 0.955) (de Mello Sa et al. 2025).

Knowledge gains were also consistent in 3 RCTs, though using non‐validated tools. Musiimenta et al. (2022) and Sosanya et al. (2025) reported higher scores; Seyyedi et al. (2021) significant knowledge/attitude gains, stronger with education or engagement. Musiimenta et al. (2022) reported increased antenatal care knowledge (OR = 3.1; *p* = 0.19) and HIV testing frequency (OR = 2.4; *p* = 0.25), though the results are not significant, they represent clinically meaningful effect sizes. Sosanya et al. (2025) found higher postintervention scores in EBF, complementary feeding, and total infant feeding knowledge (adjusted increase = 7.36; *p* = 0.000), particularly for expressed milk, maintenance, and storage (*p* < 0.05). Seyyedi et al. (2021) found significant improvements in knowledge and attitude scores in the intervention group compared with controls (5.67 ± 0.94 and 8.75 ± 1.37 respectively; both *p* < 0.001), while the increase in practice score was considered marginally significant (0.8 ± 0.49; *p* = 0.063). Effects were stronger with higher maternal education and active app engagement (Musiimenta et al. 2022). These capability improvements exceed behavioural outcomes, suggesting apps excel at psychological readiness but need complementary strategies for sustained practice.

#### 3.4.3 Early Feeding Practices and Initiation, Sustained Practices, Attitudes, and Experiences

Across the three trials reporting breastfeeding initiation, effects were generally modest, with benefits mainly confined to specific subgroups. Lewkowitz et al. (2020) reported no difference (79.5% vs. 78.6%; RR = 0.97, 95% CI 0.83–1.12; *p* = 0.60), and Uscher‐Pines et al. (2019 and 2025) showed modest overall improvements (70.6% among intervention group vs. 66.8% among in controls; adjusted difference 3.6 percentage points; *p* = 0 .08), with subgroup clear gains among Black participants (65.1% among intervention group vs. 57.4% among in controls; *p* = 0.045) and adherence‐related improvements (10.2 percentage points; *p* = 0.008). These findings suggest that mHealth may support initiation for some women, particularly marginalised groups, but is insufficient on its own to shift population‑level initiation rates.

Results for breastfeeding duration and frequency were similarly mixed. de Mello Sa et al. (2025) and Uscher‐Pines et al. (2019) observed non‐significant trends toward longer breastfeeding (68.8% among intervention group vs. 50.0% among controls at 12 months, *p* = 0.241; 71% vs. 68% at 3 months, *p* = 0.73), while Lewkowitz et al. (2020), Acar and Şahin (2024), and Vila‐Candel et al. (2024) found no clear differences in duration. In Acar and Şahin (2024), the intervention primarily reduced breastfeeding problems rather than extending duration. Taken together, these studies indicate that while apps can improve the quality of breastfeeding experiences, translating this into longer duration is challenging when work, family expectations, and health‑system constraints remain unchanged.

Attitudinal outcomes and satisfaction showed more consistent improvements, although not universally. Seyyedi et al. (2021) reported improvements in attitudes (mean 65.35, 95% CI 62.39–68.31; *p* = 0.010) and knowledge, attitude and practice (KAP) scores (Mean change = 23.57 ± 5.09; *p* < 0.001), whereas Uscher‐Pines et al. (2019) found no overall differences (ITT: 73% vs. 78%, *p* = 0.41), despite high satisfaction among video call participants (91%). This suggests that participants who actively engage with interactive features derive substantial perceived benefit, even when average attitudes and satisfaction do not change markedly at group level. This engagement‐dependent pattern reinforces that interactive mHealth features benefit active users most.

#### 3.4.4 Managing Breastfeeding Difficulties and Practices

Mobile interventions were more clearly beneficial for managing breastfeeding difficulties and perceived effectiveness. Acar and Şahin (2024) observed lower rates of nipple pain, cracked nipples, and perceived insufficient milk (*p* < 0.05), alongside higher EBF at follow‑up (86.1%–88.9% among intervention group vs. 64.9% among in controls; *p* < 0.05) and declining Breastfeeding Experience Scale scores, indicating fewer mechanical and social problems. Across studies, participants frequently valued usability, timely reassurance, and access to tailored advice, highlighting that digital tools can substantially improve day‑to‑day breastfeeding experience even when headline behavioural metrics (initiation, duration) show limited change.

Evidence on broader behavioural change beyond breastfeeding (e.g., antenatal care attendance and HIV testing) was mixed. Seyyedi et al. (2021) observed significant gains in KAP and self‐efficacy increasing by 26.85 ± 7.13 vs. +0.40 ± 5.17 in control (*p* < 0.001), whereas Musiimenta et al. (2022) reported higher odds of recommended antenatal care behaviours (OR 2.4–3.1), such as attending ANC visits and the recommended timing and frequency of HIV testing, though non‐significant (*p* = 0.14–0.25). At 6 weeks, all intervention participants reported EBF versus 81% of controls. Overall, these patterns reinforce that mHealth interventions more reliably influence proximal psychological and practical outcomes, such as confidence, attitudes, and problem‑solving than complex, multi‐determined behaviours like long‑term breastfeeding continuation.

### 3.5 Binomial Probability Test

The binomial probability test (Supporting Material 8) shows generally favourable intervention effects, though limited by small study numbers. For early EBF (Day 2 postpartum) and longer‐term breastfeeding self‐efficacy, all studies reported positive effects (100%), but with wide confidence intervals due to small samples. For outcomes assessed by multiple studies, such as EBF at 4–6 weeks postpartum and breastfeeding initiation, 66.7%–100% reported positive effects, again with wide 95% confidence intervals (0.0943–0.9916). In contrast, outcomes like breastfeeding self‐efficacy at 12 months and breastfeeding attitude/satisfaction showed fewer positive findings (0%–50%), with similarly broad intervals. Overall, most studies favoured interventions, but the small evidence base and imprecision limit firm conclusions, highlighting the need for further high‐quality research to establish consistency and effect size.

### 3.6 Meta‐Analysis

**Figure 4:**
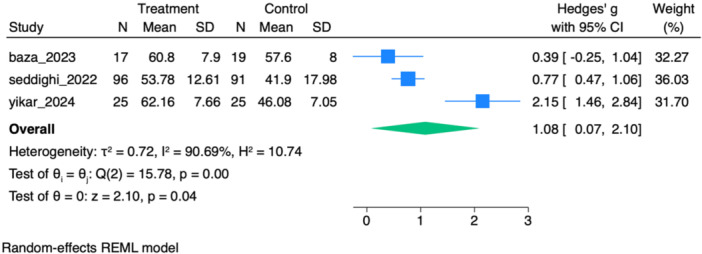
Forest plot and meta‐analysis of breastfeeding self‐efficacy. Random‐effects meta‐analysis (REML) of three studies showed a significant improvement in breastfeeding self‐efficacy (Hedges′ *g* = 1.08, 95% CI 0.07–2.10, *p* = 0.036), with high heterogeneity (*I* ^2^ = 90.7%).

Three studies (Saucedo Baza et al. 2023; Seddighi et al. 2022; Karaçay Yıkar and Nazik 2024) reporting breastfeeding self‐efficacy at 4–8 weeks were included in a random‐effects meta‐analysis using the Restricted Maximum Likelihood (REML) model. REML was used to estimate between‐study variance, providing more accurate effect estimates in the presence of heterogeneity and a small number of studies (Langan et al. 2019). The pooled effect showed significant improvement among intervention participants compared with controls (Hedges′ *g* = 1.08, 95% confidence interval (CI) 0.07–2.1; *p* = 0.036). A forest plot highlighting individual study contributions and the influence of this outlier is presented in Figure 4.

Substantial heterogeneity was observed (*I* ^2^ = 90.7%, *p* < 0.001), likely reflecting differences in population characteristics, intervention delivery, and follow‐up duration. Prediction intervals indicated that although the average effect was positive, outcomes may vary widely across settings depending on these factors. Due to high heterogeneity and a limited number of studies, we employed harvest plots to visualise results and binomial probability tests to confirm effect direction, ensuring a robust conclusion despite the limitations of the data.

## 4 Discussion

This systematic review synthesised evidence from 13 randomised controlled trials involving 3269 pregnant and postpartum women, of whom 38.5% were from low‐ and middle‐income countries. Interventions were categorised as self‐guided educational mobile applications, apps with live support, or blended models integrating tele‐support through SMS or telephone. Common features included educational modules, access to healthcare professionals, reminders, and FAQs, while fewer addressed multilingual inclusion, workplace support, or product guidance. Initiated during pregnancy or postpartum and lasting from 6 weeks to 12 months, these interventions consistently improved maternal breastfeeding knowledge and self‐efficacy, although effects on exclusive breastfeeding, initiation, duration, and behavioural practices varied. Interventions offering interactive or personalised support demonstrated the greatest overall effectiveness.

### 4.1 Effectiveness of mHealth Interventions

Self‐efficacy improvements were robust (meta‐analysis: Hedges′ *g* = 1.08, *p* = 0.036; 100% positive studies at early timepoints per binomial test), reflecting psychological mechanisms enhanced by education/reminders. In contrast, behavioral outcomes (EBF, initiation, duration) showed mixed results (66.7%–100% positive direction but wide CIs; non‐significant in most), limited by low adherence, short follow‐ups, and external barriers (e.g., work). Gains of self‐effecacy persisted up to 6 months postpartum but declined by 12 months, highlighting the need for sustained user engagement. Evidence for EBF was generally supportive but mixed: early outcomes within 3 months showed minimal benefits, while follow‐ups at 6 months demonstrated clearer improvements, particularly when adherence was maintained. Subgroup analyses revealed stronger effects among Black participants in the United States, reinforcing the importance of equity‐focused and culturally tailored approaches.

Interventions showed limited effects on breastfeeding initiation and duration, with modest improvements in more engaged subgroups. Behavioural outcomes such as maternal attitudes, satisfaction, and practical practices varied across studies, reflecting the complex influences of socio‐cultural norms, family support, and healthcare access that extend beyond digital interventions. In contrast, breastfeeding knowledge consistently improved, especially when educational content was combined with interactive features or personalised feedback. Several interventions also reduced common breastfeeding difficulties such as nipple pain and perceived milk insufficiency, indicating that mobile application–based approaches can enhance both breastfeeding effectiveness and mothers' overall experience.

### 4.2 Intervention Features, Outcomes, and COM‐B Analysis

**Figure 5:**
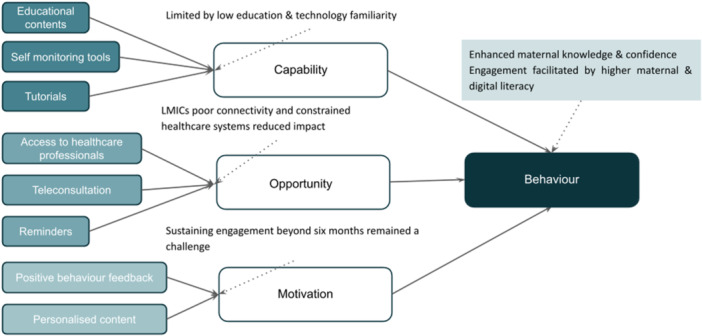
COM‐B analysis.

Mobile application‐based interventions for breastfeeding support ranged in complexity from self‐guided educational applications to interactive, personalised approaches, underscoring their multifaceted design (Ziebart et al. 2024). Interventions also target both immediate breastfeeding practices and broader outcomes, such as maternal knowledge, attitudes, and behaviours, which may support long‐term breastfeeding success and maternal satisfaction. Applying the COM‐B model highlighted key mechanisms shaping effectiveness (Figure 5, Supporting Information S1: Materials 9 and 10):

- Capability: was improved through educational content, self‐monitoring tools, and tutorials that enhanced maternal knowledge and confidence, with engagement facilitated by higher maternal and digital literacy, but limited by low education and technology familiarity.
- Opportunity: was expanded via access to healthcare professionals, and reminders, though in LMICs poor connectivity and constrained healthcare systems reduced impact.
- Motivation: was reinforced via positive behaviour feedback and personalised content, but sustaining engagement beyond 6 months remained a challenge.

Regional and socio‐cultural differences strongly shaped intervention effectiveness, underscoring the need for context‐specific tailoring (Gilano et al. 2023; Hassen 2024; Jahanpour et al. 2023). In UMIC/HICs, participants generally exhibited stronger capability due to higher knowledge and prior breastfeeding experience, greater opportunities supported by smartphone access and family encouragement, and reinforced motivation through partner and community support. In contrast, limited literacy, poverty, rural residence, and poor technological access constrained capability and opportunity in low‐ and middle‐income countries, while economic insecurity and food scarcity weakened motivation (Corkery‐Hayward and Talaei 2024). These findings highlight that interventions must strengthen literacy and knowledge to improve capability, expand technological and social access to create opportunities, and address economic and cultural barriers to sustain motivation.

Intervention design also played a crucial role. Self‐guided educational apps delivering videos, FAQs, and structured content improved maternal knowledge and self‐efficacy by consolidating capability and motivation (Chan et al. 2016; Maleki et al. 2021; Wong et al. 2021), though limited interactivity reduced engagement. Live‐support interventions, offering teleconsultation or video calls, showed stronger outcomes for knowledge, self‐efficacy, and in some cases exclusive breastfeeding (Iamchareon and Maneesriwongul 2025), facilitated by professional input and real‐time feedback yet limited by accessibility challenges. Blended approaches combining app‐based education with SMS reminders, telephone follow‐ups, or personalised feedback achieved the most comprehensive benefits by enhancing capability through multimodal learning, maintaining support opportunities, and reinforcing motivation (Hadizadeh‐Talasaz and Abdollahpour 2025). However, infrastructural constraints and declining engagement continued to hinder long‐term sustainability. Cross‐cutting features such as engagement tracking, personalised content, and infant monitoring strengthened both capability and motivation but were often moderated by cultural and systemic factors. Overall, effective mHealth interventions must integrate contextually tailored strategies that enhance capability, expand opportunity, and sustain motivation across diverse socioeconomic and cultural settings.

### 4.3 Heterogeneity Across Studies

High meta‐analytic heterogeneity (*I* ^2^ = 90.7%) and variable harvest plot effects (Figure 3) stem from differences in intervention intensity (self‐guided vs. telelactation), follow‐up duration (6 weeks–12 months), maternal parity (primiparous‐dominant in LMICs vs. multiparous in HICs), and context (urban HICs vs. rural LMICs). Interactive interventions (46.2%) outperformed static apps, with effects fading > 6 months. Primiparous women showed greater self‐efficacy gains, but EBF benefits were inconsistent due to unmeasured confounders like work return. Regional stratification (Figure 2) reveals LMIC barriers (e.g., connectivity) attenuating effects versus HICs strengths (e.g., family support).

### 4.4 Comparison With Previous Evidence

Previous systematic reviews have shown that mHealth interventions can improve maternal and neonatal outcomes in LMICs, enhancing short‐term breastfeeding initiation and exclusivity through educational content, reminders, and professional support (Sondaal et al. 2016; Daly et al. 2018; Pratiwi et al. 2023). However, these studies were limited by heterogeneity in design, intervention type, and outcome measurement, restricting comparability and the feasibility of meta‐analysis (McKay et al. 2018; Sondaal et al. 2016). While prior evidence highlighted benefits for antenatal care and perinatal outcomes, results on long‐term breastfeeding and engagement among vulnerable groups remained scarce (Diniz et al. 2019; Leal et al. 2023).

This review builds on and extends prior findings by focusing on RCTs of mobile application‐based interventions and employing standardised synthesis approaches across all income settings. Consistent with earlier reviews, our results confirm positive effects on breastfeeding initiation and exclusivity but reveal that sustained improvements depend on accessibility, usability, and socio‐cultural fit. In contrast to earlier assumptions that access alone ensures effectiveness, this review shows that engagement quality and cultural adaptation are crucial mediators. By using binomial probability analysis and harvest plots, this review provides a robust synthesis of intervention effects. Furthermore, the regional analysis reveals persistent inequities that continue to affect rural and low‐literacy populations, where barriers to digital health access remain high (Mieso et al. 2022; Qian et al. 2021; Ziebart et al. 2024; Diniz et al. 2019; Knop et al. 2024). Overall, it advances current knowledge by emphasising the need for contextually tailored, equitable, and systematically evaluated mHealth interventions to sustain breastfeeding outcomes globally.

### 4.5 Strengths and Limitations

This review has notable strengths, including a comprehensive global systematic literature search, exclusive focus on RCTs, and application of the COM‐B framework to contextualise facilitators and barriers influencing intervention effectiveness. This offers a robust synthesis of evidence on mobile application‐based interventions for breastfeeding promotion. However, several limitations are evident in the existing literature. Restricting studies to those published in English may introduce language bias (Dobrescu et al. 2021; Jüni et al. 2002), while publication bias could lead to an overestimation of effectiveness (Murad et al. 2018; Siontis et al. 2011). Apart from that, measurement inconsistency, such as validated scales for self‐efficacy and non‐validated author‐designed tools for knowledge or EBF, may inflate heterogeneity and bias pooled estimates toward null for behavioral outcomes. Standardised tools in future RCTs would enhance comparability.

The heterogeneity in intervention design, delivery, and outcomes complicates efforts to synthesise findings across studies. Many studies also suffer from small sample sizes, variable outcome measurements, and limited long‐term follow‐up, further constraining the robustness of the findings (Higgins et al. 2019c). The high heterogeneity observed in meta‐analyses warrants cautious interpretation of the results (Choi and Kang 2025). Moreover, reporting bias was not formally assessed, and the certainty of evidence for individual outcomes was not evaluated, rendering conclusions about effectiveness and generalisability provisional (Higgins et al. 2019d). Despite rigorous dual‐reviewer processes, residual reviewer bias may persist in subjective elements of study selection, data interpretation, and synthesis, particularly given the heterogeneity in study quality and reporting. Future reviews could incorporate blinded screening or automated tools to further minimise this risk.

### 4.6 Regional Differences and Generalisability

Pooling HIC/UMIC (61.5%) and LMIC (38.5%) studies enables global benchmarking but risks overgeneralisation given disparities in smartphone access (near‐universal in HICs vs. limited in LMICs), digital literacy, and cultural norms (Figure 2). Stronger self‐efficacy gains in interactive HIC apps may not translate to LMICs without offline features or low‐literacy designs. Conclusions emphasise context‐sensitive tailoring, with LMIC caution due to underrepresentation (*n* = 5 studies). Findings from US‐centric telelactation (e.g., Uscher‐Pines et al. 2019, 2025) have limited external validity for rural African or Asian settings.

### 4.7 Implications for Healthcare, Research, and Policy

Future research should prioritise developing international consensus on core outcomes for breastfeeding interventions, as current studies use heterogeneous measures that limit comparability and synthesis. Standardising outcomes would strengthen the evidence base and enable meaningful cross‐study comparisons. Culturally sensitive and participatory approaches are also essential to reflect diverse breastfeeding practices, beliefs, and barriers, ensuring women's meaningful involvement in intervention design. Furthermore, integrating multilingual support within mHealth applications remains a key gap; incorporating preferred languages and culturally appropriate terminology could improve engagement, comprehension, and equity in breastfeeding outcomes.

With only 38.5% LMIC studies, digital divide issues of low smartphone ownership, poor connectivity, low literacy, and no‐cost apps' hidden data costs, temper generalisability from HICs‐dominant evidence. Future interventions require offline functionality, voice‐based content, multilingual low‐literacy designs, and integration with community health workers. HICs telelactation success does not extend to LMICs without infrastructure, as overgeneralisation risks inequitable policy.

Policymakers and health technology developers should prioritise funding and developing interactive, personalised mobile applications that strengthen maternal breastfeeding knowledge and self‐efficacy through live support, telelactation, and individualised feedback rather than relying solely on educational content (World Health Organisation 2019). Because breastfeeding behaviours are shaped by social, cultural, and systemic factors, effective mHealth interventions must be integrated within broader healthcare frameworks that include family engagement, access to care, and local cultural norms. Sustaining engagement is critical, as early improvements often decline without continued support; thus, scalability and cost‐effectiveness should be embedded in design. Implementation success depends on literacy, language, cultural norms, technology access, and social support (Anstey et al. 2018; Asiodu et al. 2017; Bengough et al. 2022; Galvão et al. 2021; Hatami and Motamed 2012; Regan and Brown 2019). To reduce inequities, particularly in LMICs and among marginalised groups, interventions should employ simplified interfaces, multimedia and offline features, and culturally adapted messaging to improve accessibility, adherence, and breastfeeding outcomes.

## Author Contributions

Y.C.Y. conceived and designed the study, developed the methodology, and supervised the research team with consultation with K.S. and M.F. A review team consisting of Y.C.Y., M.W., P.H.L. and K.S. performed the screening and data extraction, Y.C.Y. analysed the data. Y.C.Y. drafted the initial manuscript and incorporated critical revisions suggested by all co‐authors. All authors reviewed and approved the final version of the manuscript and agreed to be accountable for all aspects of the work to ensure its accuracy and integrity.

## Conflicts of Interest

Here is to confirm no conflict of interest declared for all authors. There is no conflict of interest statement during the submission process. KS is funded by an NIHR Doctoral Fellowship (NIHR302577). The views expressed are those of the author(s) and not necessarily those of the NIHR or the Department of Health and Social Care.

## Supporting information


Supporting File 1

Supporting File 2

Supporting File 3

Supporting File 4

Supporting File 5

Supporting File 6

Supporting File 7

Supporting File 8

Supporting File 9

Supporting File 10

## Data Availability

The data that support the findings of this study are available from the corresponding author upon reasonable request.
