# Supplementary material for: Evaluating the Effectiveness of mHealth Application Interventions to Promote Breastfeeding Amongst Pregnant Women and Postpartum Mothers: A Global Systematic Review and Meta‐Analysis
Source: Matern Child Nutr. 2026 Aug 17;22(3):e70230. doi: 10.1111/mcn.70230 (PMC13480556; doi:10.1111/mcn.70230)
Supplement: Supplementary file 1 [file MCN-22-e70230-s001.docx]

**Supplementary Materials 10**. Summary of Digital Intervention Types, Key Features, and Clinical Outcomes Mapped to COM-B Behavioral Determinants.

| Intervention Type | Key Features | Primary Outcomes | Facilitators (COM-B) | Barriers (COM-B) |
| --- | --- | --- | --- | --- |
| Self-guided educational apps | Online educational content, videos, FAQs | ↑ Maternal knowledge, ↑ Self-efficacy | Capability: Accessible, structured content Motivation: Knowledge reinforcement | Opportunity: Limited interactivity Motivation: Less engagement over time |
| Apps with live support | Direct access to healthcare professionals, teleconsultation, video calls | ↑ Self-efficacy, ↑ Knowledge, ↑ EBF | Capability: Personalised guidance Opportunity: Real-time support Motivation: Feedback and reinforcement | Opportunity: Limited access in LMICs Motivation: Adherence dependent |
| Blended interventions (app + SMS/phone support) | App content + reminders, telephone follow-up, personalised feedback | ↑ Knowledge, ↑ Self-efficacy, ↓ breastfeeding difficulties | Capability: Multi-modal learning Opportunity: Continuous support Motivation: Personalised encouragement | Opportunity: Infrastructure challenges in LMICs Motivation: Engagement drop-off over long follow-up |
| All intervention types | Engagement tracking, personalised content delivery, infant monitoring tools | Mixed effects on initiation, duration, behavioural practices | Capability: Enhanced through structured guidance Motivation: Self-monitoring tools | Opportunity: Cultural, familial, systemic factors limiting behaviour change Motivation: Sustaining long-term engagement |
