# Supplementary material for: Evaluating the Effectiveness of mHealth Application Interventions to Promote Breastfeeding Amongst Pregnant Women and Postpartum Mothers: A Global Systematic Review and Meta‐Analysis
Source: Matern Child Nutr. 2026 Aug 17;22(3):e70230. doi: 10.1111/mcn.70230 (PMC13480556; doi:10.1111/mcn.70230)
Supplement: Supplementary file 2 [file MCN-22-e70230-s002.docx]

**Supplementary Material 6**. RoB 2 assessment and CONSORT adherence.

Note: The RoB 2 tool, developed by Cochrane, assesses risk of bias in randomised controlled trials, while the CONSORT statement provides evidence-based guidelines to enhance their transparency and reporting quality (Cochrane Methods Bias, 2025; Cuschieri, 2019). As reached consensus for all review panel members, for the RoB2 Q1-5 assessment each criterion was evaluated as “Yes,” “Probably Yes,” “Probably No,” “No” or “No information”; a rating of two “Probably No” responses or any “No information” was classified as “Some Concerns” regarding risk of bias.

Overall Risk of Bias Summary bar chart.

RoB2 assessment chart and adherence to CONSORT statement.
