# Supplementary material for: Evaluating the Effectiveness of mHealth Application Interventions to Promote Breastfeeding Amongst Pregnant Women and Postpartum Mothers: A Global Systematic Review and Meta‐Analysis
Source: Matern Child Nutr. 2026 Aug 17;22(3):e70230. doi: 10.1111/mcn.70230 (PMC13480556; doi:10.1111/mcn.70230)
Supplement: Supplementary file 3 [file MCN-22-e70230-s003.docx]

**Supplementary Material 1.** Method Details.

Literature Search

This systematic review is reported in accordance with the Preferred Reporting Items for Systematic reviews and Meta-Analyses (PRISMA, 2025) guidelines. The search strategy was based on the Population, Intervention, Comparator, Outcome framework (**Supplementary Material 1 and 2**) and structured around three root concepts: breastfeeding, mHealth/mobile interventions, and study design, incorporating relevant Medical Subject Headings (MeSH), truncations, synonyms, and alternate spellings, combined with ‘OR’ operators. The literature search was conducted from 1st January 2010 to 1st May 2025 across PubMed/MEDLINE, Embase, CINAHL, Web of Science, and the Cochrane Library. The 2010 start date aligns with the World Health Organisation's formal definition and global expansion of mHealth technologies (WHO, 2011), marking the proliferation of smartphone apps and digital breastfeeding support following the iPhone launch (2007) and Android adoption. This ensures focus on contemporary, scalable interventions relevant to current breastfeeding guidelines (WHO, 2024). The databases were selected for their comprehensive coverage of biomedical, nursing, allied health, and multidisciplinary literature relevant to mHealth and breastfeeding interventions. PubMed and Embase provide extensive indexing of clinical trials and health technology studies; CINAHL specialises in nursing and maternal-child health; Web of Science captures multidisciplinary and emerging digital health research; and Cochrane ensures inclusion of high-quality systematic reviews. Collectively, they encompass global peer-reviewed literature in English, with hand-searching of reference lists supplementing grey literature gaps. All references managed in Rayyan (2025) and duplicates removed prior to screening.

Inclusion and Exclusion criteria
Details of Interventions targeting pregnant women or mothers up to 12 months postpartum who intended to breastfeed, irrespective of parity or infant health (including term or preterm infants), were eligible. The primary outcome of interest was exclusive breastfeeding (EBF) up to 6 months, defined in accordance with WHO (2024) as breast milk only for the first 6 months of life, while secondary outcomes included EBF at later time points, any breastfeeding rates, overall breastfeeding duration, and breastfeeding self-efficacy up to 12 months. The EBF outcome and secondary outcomes in both the narrative synthesis and the harvest plots (**Figure 3**) are analysed separately, distinguishing EBF outcome and other secondary outcomes measured beyond 6 months. mHealth interventions delivering educational content, peer support, reminders, or telehealth consultations were included. Only randomised controlled trials (RCTs) published in English were eligible and were required to report at least one breastfeeding-related outcome (initiation, exclusivity, duration, maternal knowledge, attitudes, self-efficacy, or app engagement). Studies targeting health professionals or family members, using solely face-to-face or hybrid approaches without a mobile component, employing non-randomised or qualitative designs, or lacking relevant breastfeeding outcomes were excluded.

Selection Process and Data Extraction

Two independent reviewers (CY and MW/LP/KS) screened abstracts and full texts using a standardised checklist based on pre-defined inclusion/exclusion criteria (Table 2). Disagreements were resolved through discussion or consultation with a third reviewer to minimise selection bias. Data extraction was performed independently by two reviewers using a piloted form, with cross-checking and consensus to reduce extraction errors. Narrative synthesis followed Cochrane guidance, categorising interventions and outcomes with input from the research team and experts by experience to mitigate synthesis bias. Inter-rater reliability was not formally quantified but achieved through iterative calibration meetings prior to screening. Outcome variability was addressed via standardised extraction and narrative grouping; non-validated tools were flagged in risk of bias.

Risk of Bias Assessment

Risk of bias was assessed using the Cochrane Risk of Bias Tool version 2 (RoB-2) (Cochrane Methods Bias, 2025; Higgins et al., 2019). This covered randomisation, deviations from intended interventions, missing outcome data, outcome measurement, and selective reporting. Reporting quality was evaluated via the CONSORT checklist (Cuschieri, 2019). Two reviewers conducted assessments independently, resolving disagreements through discussion or third reviewer consultation.

Data Synthesis

Narrative synthesis was conducted according to Cochrane guidance and included creation of categories of interventions, focusing on intervention type, breastfeeding outcomes, and context with input from the research team and experts by experience (Higgins et al., 2025). A meta-analysis was conducted if sufficient studies reported data on the same outcomes in a format that could be meaningfully combined into estimates of pooled odds ratios or risk ratios. Pooled effect sizes with 95% confidence and prediction intervals were calculated in STATA version 19 using random-effects models to account for heterogeneity (DerSimonian & Laird, 1986; STATA, 2025), with statistical significance set at P <0.05. Heterogeneity was evaluated via the I² statistic, with I² > 50% indicating substantial heterogeneity (Higgins et al., 2003). Cochrane guidance was followed to create a Harvest Plot (Higgins et al., 2025) due to substantial heterogeneity in outcomes. A Harvest Plot is a visual supermatrix summarising vote counting by categorising studies as having a beneficial effect or no effect in each outcome category, where each bar represents a single study annotated with the reference number, and bar height indicates study quality: strong (tallest), moderate (medium), or weak (shortest). In accordance with Cochrane guidance, the proportion of beneficial studies using binomial probability tests was calculated in R (Higgins et al., 2025), the number of studies showing improvement compared to the total outcomes reported per intervention category, were analysed. Publication bias via funnel plots or Egger’s test (Egger et al., 1997) was assessed. Studies from HICs/UMICs and LMICs were analysed together to provide a global overview of mHealth effectiveness, stratified by region in narrative synthesis, harvest plots, and binomial tests.
