# Supplementary material for: Evaluating the Effectiveness of mHealth Application Interventions to Promote Breastfeeding Amongst Pregnant Women and Postpartum Mothers: A Global Systematic Review and Meta‐Analysis
Source: Matern Child Nutr. 2026 Aug 17;22(3):e70230. doi: 10.1111/mcn.70230 (PMC13480556; doi:10.1111/mcn.70230)
Supplement: Supplementary file 4 [file MCN-22-e70230-s004.docx]

**Supplementary Material 7**. Summary of key facilitators and barriers by countries.

| **Domains** | **Upper-and Middle-Income and High-Income Countries (UMICs and HICs)** | **Low- and Middle-Income Countries (LMICs)** | **Facilitators / Barriers** |
| --- | --- | --- | --- |
| Age / Parity | Older mothers (22-34y), mixed parity | Younger mothers (15–27y), often primiparous | Barrier: First-time mothers may need more support;  Facilitator: Experienced mothers may engage better |
| Education / Literacy | Mostly high school or college; high literacy | Mostly primary/secondary; literacy limitations | Barrier: Low literacy hinders app use;  Facilitator: High literacy improves comprehension |
| Employment / Income | 45–60% employed; moderate income | Most unemployed or informal work; very low income | Barrier: Financial constraints limit access;  Facilitator: Employed mothers may have autonomy and resources |
| Marital / Family Support | 32–80% married/cohabiting | 83–95% married/cohabiting | Barrier: Lack of support reduces engagement;  Facilitator: Partner/family support enhances adherence |
| Technology Access | High smartphone ownership (89–97%) | Limited device access | Barrier: No device or limited connectivity;  Facilitator: Widespread smartphone use enables mHealth delivery |
| Pregnancy / Delivery | Mostly planned pregnancies; C-section 15–47% | Planned pregnancies moderate; C-section 25–75% | Barrier: Cesarean may delay breastfeeding;  Facilitator: Planned pregnancy encourages engagement |
| Prior Breastfeeding Experience | 56–75% prior experience | Often first-time mothers | Barrier: No prior experience may reduce confidence;  Facilitator: Experienced mothers engage more effectively |
| Geography / Access to Care | Urban, moderate travel | Rural, long distances (8–20 km) | Barrier: Distance limits facility support;  Facilitator: mHealth applications can bridge access gaps |
| Cultural Factors | Multiethnic; language not a major barrier | Homogenous; local language critical | Barrier: Language and cultural mismatch;  Facilitator: Culturally tailored content increases uptake |
