# Supplementary material for: Evaluating the Effectiveness of mHealth Application Interventions to Promote Breastfeeding Amongst Pregnant Women and Postpartum Mothers: A Global Systematic Review and Meta‐Analysis
Source: Matern Child Nutr. 2026 Aug 17;22(3):e70230. doi: 10.1111/mcn.70230 (PMC13480556; doi:10.1111/mcn.70230)
Supplement: Supplementary file 5 [file MCN-22-e70230-s005.docx]

**Supplementary Material 4**. Full study details on interventions and outcomes.

| **First author¹, year** | **Country** | **Study Population** | **Perinatal Period** | **Intervention details** | **Intervention settings****** | **Study Population*** **(age mean)** | **Intervention subjects, n**** | **Control subjects, n**** | **Comparison group²** | **Relevant Measured Outcomes** | **Outcome measurement** | **Result**  **Summary³******* |
| --- | --- | --- | --- | --- | --- | --- | --- | --- | --- | --- | --- | --- |
| (1) Self-guided mobile applications providing educational content and breastfeeding resources | | | | | | | | | | |  |  |
| de Mello Sa, 2025 | United States | Primiparous and multiparous females | Antenatal to postnatal | Breastfeeding at AU used throughout the first year; educational, timelines, FAQ, and institutional resources | Pr to PP****,  Home, Community | PW* (33.4) | 16 | 20 | Standard Care (SC) | 1. Breastfeeding duration  2. EBF at 6 months postpartum  3. Change in BSES-SF⁴ total score (self-efficacy) | Self-reported surveys, BSES-SF⁴  Baseline (prenatal): Between 32nd–36th weeks of gestation  Follow-up: 6 mths + 1 year postpartum | Duration: 10.1 vs. 8.9 months (NS*****).  6-mo EBF: 81.3% vs. 60.0% (NS).  A Kaplan–Meier survival curve demonstrated a higher proportion of participants who breastfed for longer durations in the intervention group (p = .241) (NS).  Feedback: Feeding trackers, real-time support, peer groups valued. |
| Lewkowitz, 2020 | United States | First-time mothers | Antenatal to postnatal | Breastfeeding Friend app with tailored educational videos/content, usage tracked | Pr to PP,  Home | PW (22.7) | 84 | 85 | Control app | 1. EBF at postpartum day 2  2. Breastfeeding  initiation and exclusive and nonexclusive breastfeeding at 6 weeks, 3 months, and 6 months postpartum  3. Reported breastfeeding challenges and reasons for formula initiation  4. Reported best breastfeeding or pumping resources both in the hospital and at home | Self-reported surveys, Infant Feeding Practices Study II questionnaire | Day 2 EBF: 36.6% (BF Friend) vs. 35.7% (control); RR = 1.02 (95% CI 0.68–1.53).  6-mo EBF: 8.3% vs. 10.4%; RR = 0.80 (95% CI 0.27–2.38).  6-wk support: 52.3% BF Friend users rated app best support.  Non-users: 21.4% (BF Friend) vs. 10.6% (control); exclusion did not alter outcomes. |
| Baza, 2023 | United States | Mothers | Postnatal | Breastfeeding at AU used through 4–6 weeks postpartum | PP, Home | PM (24.5) | 17 | 19 | Usual Care (UC) | 1. Breastfeeding self-efficacy  2. EBF rates at 4-6 weeks postpartum  3. Breastfeeding duration  4. Breastfeeding continuation plans | Self-reported surveys, BSES-SF⁴ | BSES-SF⁴: Increased 7.6 points in intervention (p < 0.001).  4–6 wk EBF: 65% vs. 32%; positive trend (p = 0.093) (S*****).  Continuation plans: 94% of intervention participants intended to continue breastfeeding.  App usability: 100% easy to navigate; 88% found useful.  Self-efficacy change: Intervention M = 7.6 (SD 7.8) vs. control M = 1.2 (SD 3.7), p = .001.  EBF rate nearly double in intervention (NS). |
| Acar, 2024 | Turkey | Primiparous mothers | Postnatal | MABBP app with educational content, logging tools, and motivational notifications | PP, Home | PM (27.7) | 36 | 37 | SC | 1. Breastfeeding effectiveness and difficulties  2. Breastfeeding frequency and duration  3. Breastfeeding problems and reasons for interruption | Infant Breastfeeding Assessment Tool and Breastfeeding Experience Scale, researcher-developed follow-up forms for breastfeeding history | Breastfeeding problems: Decreased at first follow-up 44.4% vs 73.0% (p = 0.014), second follow-up 22.2% vs 67.6% (p < 0.001).  Pain/cracked nipples: 0% vs 13.5% at second follow-up (p = 0.029).  Exclusive breastfeeding: Increased first follow-up 86.1% vs 64.9% (p = 0.035), second follow-up 88.9% vs 64.9% (p = 0.015).  BES total score: Decreased first day 38.05 ± 9.69 → second follow-up 30.3 ± 7.44 (p = 0.002); within-group improvement p < 0.001. |
| Seddighi, 2022 | Iran | Mothers | Postnatal | An 8-week app delivering breastfeeding education | PP, Home or community | PM* (29.8) | 96 | 91 | SC | Breastfeeding self-efficacy | BSES-SF⁴ | Increased breastfeeding self-efficacy (mean difference = 5.52, SD = 12.35; p < 0.001). |
| (2) Mobile applications combined with live support | | | | | | | | | | |  |  |
| Musiimenta, 2022 | Uganda | Pregnant women | Antenatal to postnatal | MatHealth app includes access to an obstetrician and multimedia content | Pr to PP, Home | PW (27) | 40 | 40 | SC | Maternal and children health-related knowledge and practice | Self-reported surveys with multiple-choice, yes/no, and open-ended questions | 6-wk EBF: 32/32 (100%) in intervention, p = 0.01.  Knowledge outcomes: Improved numerically NS.  Other health behaviors (partner escort, facility delivery, ANC attendance): Positive trends, NS |
| Uscher-Pines, 2019 | United States | Postpartum women | Postnatal | Pacify Health app with on-demand lactation Consultants video calls | PP, Hospital, Home | PM (26.5) | 94 | 93 | UC | 1. Any breastfeeding at 3 months postpartum  2. EBF rate at 3 months  3. Breastfeeding satisfaction during the period of breastfeeding.  4. Feasibility, satisfaction with telelactation video calls among intervention participants  5. Breastfeeding self-efficacy (baseline)  6. Breastfeeding problems/challenges  7. Types of breastfeeding support received  8. Plans related to returning to work | Self-reported surveys, National Immunisation Survey | 12-wk any breastfeeding: Telelactation 71% vs. control 68% (ITT, Δ 3%, p = .73); 73% vs. 68% (IV⁵, Δ 5%, p = .74) (NS).  12-wk exclusive breastfeeding: Telelactation 51% vs. control 46% (ITT, Δ 5%, p = .47); 56% vs. 45% (IV⁵, Δ 11%, p = .48) (NS). |
| Uscher-Pines, 2025 | United States | Pregnant mothers | Antenatal to postnatal | Expanded Pacify Health version with 24/7 telelactation through 6 months postpartum | Pr to PP, Home or community | PW (***) | 955 | 956 | UC | 1. Any breastfeeding in the past 7 days  2. EBF rates at 6 months postpartum  3. Age (in months) when breastfeeding ceased (duration)  4. Breastfeeding uptake, birth experiences, sources of breastfeeding support assessed through online surveys at enrollment, 4 weeks, and 6 months postpartum | Self-reported surveys | Strongest effects: Black parents; statistically significant improvements in any & exclusive breastfeeding (both ITT & IV⁵).  Full sample: Significant only in IV⁵ models; Latinx & non-Black/non-Latinx groups, NS.* |
| Yikar, 2024 | Turkey | Pregnant women | Postnatal | Live chat available with researchers alongside app content and FAQs | PP, Home | PM (***) | 25 | 25 | E-mobile group, UC | Breastfeeding self-efficacy at 1st day and 3rd day and the 1st week, 2nd week, 3rd week, 4th week, 5th week, 6th week, and 7th week postpartum | BSES-SF⁴ | Breastfeeding self-efficacy: Increased in E-mobile training vs. control (Day 3 → Week 7, p < .001).  Between-group differences: Significant Weeks 2–7 (p < .001). |
| Abadi, 2024 | Iran | Primiparous mothers | Postnatal | App plus access to a virtual support group, expert support for 6 months. | PP, Hospital, Home | PM (28) | 20 | 20 | SC | 1. Breastfeeding self-efficacy (baseline and 6 months post-discharge)  2. Breastfeeding status monitored monthly up to 6 months | Dennis' validated breastfeeding self-efficacy questionnaire | Increased breastfeeding self-efficacy at 6 months (mean 55.80 vs. baseline 33.75; p < 0.001).  Increased breastfeeding self-efficacy in the intervention group (mean increase 22.05; p < 0.001). |
| (3) Blended interventions involving mobile apps with additional telesupport | | | | | | | | | | |  |  |
| Vila-Candel, 2024 | Spain | Pregnant women | Antenatal to postnatal | LactApp with personalised automated support, email follow-ups, and access to educational programs | Pr to PP, Community | PW (33.2) | 39 | 125 | UC | 1. EBF rate, supplementary feeding, and mixed feeding at discharge, 15 days, 6 weeks, 3 months, and 6 months postpartum  2. Breastfeeding abandonment rates over the same periods | Self-reported surveys and routine visits | Logistic regression found no significant predictors of breastfeeding abandonment across time points (all p>0.05).  The intervention group showed favorable but non-significant effects early on (discharge: Exp(B)=610,850,900.5, p=0.998; 15 days: Exp(B)=86,134,989.1, p=0.998; neutral effects mid-term (6 weeks: Exp(B)=1.343, p=0.667; 3 months: Exp(B)=1.168, p=0.763), and slightly reduced odds at 6 months (Exp(B)=0.862, p=0.699). |
| Seyyedi, 2021 | Iran | First-time mothers | Postnatal | 3-month educational app + weekly follow-up calls by midwives | PP, Home or community | PM (26) | 40 | 40 | UC | 1. Breastfeeding self-efficacy  2. Pre-post knowledge, attitudes, and practice (KAP) related to breastfeeding  3. breastfeeding-related behavioural changes | Self-reported KAP surveys, BSES-SF⁴ | Breastfeeding Knowledge: Mean 20.23 (95% CI 19.27–21.19), p < 0.001; between-group change 7.07 vs 1.40, difference 5.67, p < 0.001.  Breastfeeding Attitudes: Mean 65.35 (95% CI 62.39–68.31), p = 0.010; change 14.80 vs 6.05, difference 8.75, p < 0.001.  Breastfeeding Practice: Mean 9.13 (95% CI 8.70–9.56), p = 0.033.  Overall KAP Score: Mean 94.70 (95% CI 91.38–98.02), p < 0.001; change 23.57 vs 8.35, difference 15.22, p < 0.001.  Breastfeeding Self-Efficacy (3-mo follow-up): Mean 145.92 vs control 125.50, p < 0.001; change 26.85 vs 0.40, difference 26.45, p < 0.001. |
| Sosanya, 2025 | Nigeria | Mothers | Postnatal | BabyThrive educational app + monthly phone reminders | PP, Home | PM (***) | 97 | 97 | SC | Maternal Infant and young child feeding knowledge | Teen Moms Child Feeding Questionnaire | Knowledge Improvements Post-Intervention*.  Mean knowledge scores (BabyThrive vs Control):  EBF: 25.17 ± 1.37 vs 19.87 ± 1.80, p < 0.01.  Complementary feeding: 15.68 ± 0.60 vs 13.51 ± 1.21, p < 0.01, Total child feeding knowledge: 46.83 ± 1.79 vs 38.65 ± 2.71, p < 0.01. Regression analysis: intervention increased knowledge scores, effect sizes β = 0.71–8.19, all p = 0.000. |

*PW= Pregnant women, PM= Postpartum mothers

**Intervention and control group participants numbers, not including those who are lost to follow up or excluded for analysis

***Mean age not reported in the study

****Pr=Perinatal, PP=Postnatal

*****NS=Not significant

¹All included studies on the table are Randomised Control Trials (RCTs); Baza (2023), Musiimenta (2022) and Uscher-Pines (2019) are pilot RCT studies.

²For the comparison group column, ‘usual care’ (UC) and ‘standard care’ (SC) are delivered verbatim from the studies, which the control group has no exposure to the intervention.

³Due to wordings, here presented results summary into this table only. For statistical details please refer to original studies.

⁴BSES-SF=Breastfeeding Self-Efficacy Scale-Short Form

⁵IV (instrumental variables) adjusts ITT (intent-to-treat) analysis for treatment contamination, estimating effects on actual telelactation users only via 2SLS (randomization as instrument) (Uscher-Pines et al, 2019; Uscher-Pines et al, 2025).
