# Supplementary material for: Evaluating the Effectiveness of mHealth Application Interventions to Promote Breastfeeding Amongst Pregnant Women and Postpartum Mothers: A Global Systematic Review and Meta‐Analysis
Source: Matern Child Nutr. 2026 Aug 17;22(3):e70230. doi: 10.1111/mcn.70230 (PMC13480556; doi:10.1111/mcn.70230)
Supplement: Supplementary file 6 [file MCN-22-e70230-s006.docx]

**Supplementary Material 2**. Main features of the search strategy.

| Population | Intervention | Comparator | Outcome |
| --- | --- | --- | --- |
| Pregnant women and postpartum (up to one year) mothers | mHealth interventions delivered via mobile applications, smartphones, text messaging (SMS), telemedicine, eHealth, web-based, or other digital platforms | Standard care, usual practice, or alternative non-mHealth interventions | Breastfeeding initiation, exclusivity, duration, self-efficacy, knowledge, satisfaction, and related behavioural changes or practices |

String terms used in the final search strategy.

| Root Terms | Breastfeeding | AND | mHealth/ Mobile Interventions | AND | Study Design |
| --- | --- | --- | --- | --- | --- |
| Synonyms | Breastfeeding, exclusive breastfeeding |  | Mobile Applications, Telemedicine, Text Messaging, Cell Phone, Smartphone, mHealth, eHealth, mobile, portable software application, tele, e-health, m-health, phone, text, short message, SMS, app, apps, app-based, electronic, message, web, web-based, internet*, digital |  | Randomised controlled trial, controlled clinical trial, randomised, placebo, clinical trials, randomly, trial |
