# Supplementary material for: Evaluating the Effectiveness of mHealth Application Interventions to Promote Breastfeeding Amongst Pregnant Women and Postpartum Mothers: A Global Systematic Review and Meta‐Analysis
Source: Matern Child Nutr. 2026 Aug 17;22(3):e70230. doi: 10.1111/mcn.70230 (PMC13480556; doi:10.1111/mcn.70230)
Supplement: Supplementary file 7 [file MCN-22-e70230-s007.docx]

**Supplementary Material 9**. Synthesis of Breastfeeding Barriers and Facilitators across High-, Middle-, and Low-Income Countries mapped to the COM-B Framework.

| **Study (Year, Country)** | **Capability (Knowledge & Skills)** | **Opportunity (Physical & Social)** | **Motivation (Reflective & Automatic)** | **Barriers** | **Facilitators** |
| --- | --- | --- | --- | --- | --- |
| HICs studies | | | | | |
| de Mello Sa, 2025 (US) | High literacy; prior BF experience; moderate BSES-SF (~52) | High smartphone ownership; partner/family support; hospital environment | Reflective: planned pregnancies; Automatic: app reminders | First-time mothers or low confidence may struggle | Partner/family support and reminders enhance engagement |
| Lewkowitz, 2020 (US) | Moderate knowledge; some with <HS education; high smartphone ownership | Urban setting; peer/family support | Motivation supported by prior experience; reminders via app | Some mothers declined to answer questions, limiting engagement | Peer/family support; reminders sustained engagement |
| Uscher-Pines, 2019 (US) | Moderate knowledge; 56–57% prior BF experience | High smartphone ownership; income variability; insurance coverage | Reflective: planned BF; Automatic: notifications | Hypertension, depression/anxiety may reduce engagement; hospital stay limits | Insurance coverage; notifications supported BF intentions |
| Baza, 2023 (US) | Moderate knowledge; antenatal BSES-SF 55–59 | High smartphone access; urban setting | Planned BF; app-based reminders | Mixed education levels; some first-time mothers | Strong antenatal confidence; reminders supported engagement |
| Uscher-Pines, 2025 (US) | Moderate–high knowledge; literacy adequate | Smartphone ownership high; urban/suburban/rural mix | Planned BF; reminders | Some ethnic minorities may need culturally tailored support | Widespread smartphone ownership supports reach |
| Vila-Candel, 2024 (Spain) | High knowledge; 33–55% university education | Employment 25–75%; hospital support | Prior BF experience; planned BF | Multiparous mothers may face logistic challenges | Prior BF experience |
| UMICs studies | | | | | |
| Yikar, 2024 (Turkey) | Literacy varies; mostly primary/secondary education | Non-working mothers; family support | Reflective motivation high (planned pregnancy) | Low employment limits financial autonomy | Family support aids engagement |
| Acar, 2024 (Turkey) | Moderate knowledge; HS/university education | Employment moderate; urban settings | Moderate motivation; some prior BF experience | Small % without prior BF experience | Urban access and education enhance uptake |
| LMICs studies | | | | | |
| Musiimenta, 2022 (Uganda) | Low literacy; mostly primary; limited prior BF experience | No regular income; food insecurity; rural; spouse decision-making | Motivation constrained by financial stress; peer/family influence | Food insecurity; rural travel burden; low autonomy | App in local language (Runyankole); reduced travel burden |
| Sosanya, 2025 (Nigeria) | Low–moderate knowledge; 72% secondary education | Low income; rural; household support | Planned pregnancies; family support | Low household income; child age range 0–23 mo affects consistency | Strong family support |
| Seyyedi, 2021 (Iran) | Moderate knowledge; 30–38% diploma/academic | Mostly housewives; variable income; city/rural mix | Mixed motivation; some income stress | Cost > income for 43–55%; rural residence affects access | Some financial stability in urban areas |
| Seddighi, 2022 (Iran) | Moderate knowledge; ~40–47% university education | Housewife majority; urban/rural mix | Planned BF; moderate income | High C-section rates (71–75%) reduce early BF initiation | Higher education level supports knowledge |
| Abadi, 2024 (Iran | Moderate knowledge; 45% diploma/bachelor; 10–25% Master+ | Mostly employed; city/village distribution | Mixed; husband support 55–75% | Rural residency; low husband support for some | Husband support; employment improves opportunity |
