# Supplementary material for: Evaluating the Effectiveness of mHealth Application Interventions to Promote Breastfeeding Amongst Pregnant Women and Postpartum Mothers: A Global Systematic Review and Meta‐Analysis
Source: Matern Child Nutr. 2026 Aug 17;22(3):e70230. doi: 10.1111/mcn.70230 (PMC13480556; doi:10.1111/mcn.70230)
Supplement: Supplementary file 8 [file MCN-22-e70230-s008.docx]

**Supplementary Material 8**. Binomial probability test of Outcome Events.

Note: *Positive = intervention favored over control.

| **Outcome measure (postpartum)** | **No. of studies** | **No. positive*** | **% Positive*** | **95% Confidence interval** (rounded to three decimal places) | **p-value** |
| --- | --- | --- | --- | --- | --- |
| EBF (Day 2) | 1 | 0 | 0% | 0.000–0.975 | 1.000 |
| EBF (4–6 weeks) | 3 | 2 | 66.7% | 0.094–0.992 | 1.000 |
| EBF (3 months) | 1 | 1 | 100% | 0.025–1.000 | 1.000 |
| EBF (6 months) | 3 | 2 | 66.7% | 0.094–0.992 | 1.000 |
| Breastfeeding self‑efficacy (Day 1 and Day3) | 1 | 1 | 100% | 0.025–1.000 | 1.000 |
| Breastfeeding self‑efficacy (Week 1–3) | 1 | 1 | 100% | 0.025–1.000 | 1.000 |
| Breastfeeding self‑efficacy (4–6 weeks) | 2 | 2 | 100% | 0.158–1.000 | 0.500 |
| Breastfeeding self‑efficacy (7–8 weeks) | 2 | 2 | 100% | 0.158–1.000 | 0.500 |
| Breastfeeding self‑efficacy (3 months) | 1 | 1 | 100% | 0.025–1.000 | 1.000 |
| Breastfeeding self‑efficacy (6 months) | 1 | 1 | 100% | 0.025–1.000 | 1.000 |
| Breastfeeding self‑efficacy (12 months) | 1 | 0 | 0% | 0.000–0.975 | 1.000 |
| Breastfeeding initiation | 3 | 2 | 66.7% | 0.094–0.992 | 1.000 |
| Knowledge (maternal/breastfeeding) | 3 | 3 | 100% | 0.292–1.000 | 0.250 |
| Breastfeeding duration and frequency | 5 | 2 | 40% | 0.053–0.853 | 1.000 |
| Breastfeeding attitude/satisfaction | 2 | 1 | 50% | 0.013–0.987 | 1.000 |
| Breastfeeding effectiveness, reduced difficulties/challenges | 1 | 1 | 100% | 0.025–1.000 | 1.000 |
| Breastfeeding‑related behavioral changes/practices | 2 | 1 | 50% | 0.013–0.987 | 1.000 |
