# Supplementary material for: Evaluating the Effectiveness of mHealth Application Interventions to Promote Breastfeeding Amongst Pregnant Women and Postpartum Mothers: A Global Systematic Review and Meta‐Analysis
Source: Matern Child Nutr. 2026 Aug 17;22(3):e70230. doi: 10.1111/mcn.70230 (PMC13480556; doi:10.1111/mcn.70230)
Supplement: Supplementary file 9 [file MCN-22-e70230-s009.docx]

**Supplementary Material 3**. Search terms.

1. PubMed

#1 “exclusive breastfeeding”[Title/Abstract]

#2 “breastfeeding”[Title/Abstract]

#3 “pregnant women”[MeSH] OR “pregnant”[Title/Abstract] OR “antenatal”[Title/Abstract] OR “prenatal”[Title/Abstract]

#4 “postpartum period”[MeSH] OR “postpartum”[Title/Abstract] OR “postnatal”[Title/Abstract] OR “new mother*”[Title/Abstract]

#5 “Mobile Applications”[MeSH]

#6 “Telemedicine”[MeSH]

#7 “Text Messaging”[MeSH]

#8 “Cell Phone”[MeSH]

#9 “Smartphone”[MeSH]

#10 mobile OR “Portable Software Application” OR “Tele*” OR “mHealth” OR “eHealth” OR “e-health” OR “m-Health” OR “phone*” OR “Text*” OR “Short Message” OR “SMS” OR “app” OR “apps” OR “app-based” OR “electronic” OR “Message*” OR “web” OR “web-based” OR “Internet*” OR “digital*”[Title/Abstract]

#11 randomised controlled trial[pt] OR controlled clinical trial[pt] OR randomised[tiab] OR placebo[tiab] OR clinical trials as topic[mesh:noexp] OR randomly[tiab] OR trial[ti]

#12 animals [mh] NOT (humans [mh] AND animals [mh])

#13 #1 OR #2

#14 #3 OR #4 OR

#15 #5 OR #6 OR #7 OR #8 OR #9 OR #10

#16 #(13 AND #14 AND 15 AND #11) NOT #12

2. Embase

1 exp Breastfeeding/

2 exp pregnancy/ OR exp pregnant woman/ OR exp antenatal care/ OR exp prenatal care/ OR exp pregnant/.ti,ab,kf.

3 exp postpartum period/ OR exp puerperium/ OR exp postnatal care/ OR exp postpartum/ OR exp new mother/:ti,ab,kw

4 exp mobile application/ OR exp telemedicine/ OR exp text messaging/ OR exp mobile phone/ OR exp smartphone/:ti,ab,kw

5 exp mobile:ti,ab,kw OR exp portable software application/:ti,ab,kw OR tele*:ti,ab,kw OR mhealth:ti,ab,kw OR ehealth:ti,ab,kw OR e-health:ti,ab,kw OR m-health:ti,ab,kw OR phone*:ti,ab,kw OR text*:ti,ab,kw OR 'short message':ti,ab,kw OR sms:ti,ab,kw OR app:ti,ab,kw OR apps:ti,ab,kw OR 'app-based':ti,ab,kw OR electronic:ti,ab,kw OR message*:ti,ab,kw OR web:ti,ab,kw OR 'web-based':ti,ab,kw OR internet*:ti,ab,kw OR digital* OR digital health:ti,ab,kw

6 ('randomised controlled trial'/exp OR 'controlled clinical trial'/exp OR 'randomised':ti,ab,kw OR placebo*:ti,ab,kw OR 'double blind procedure'/exp OR 'single blind procedure'/exp OR random*:ti,ab,kw OR blind*:ti,ab,kw OR allocat*:ti,ab,kw OR trial:ti,ab,kw)

7 #2 OR #3

8 #4 OR #5

9 #1 AND #7 AND 8 AND #6

3. Web of Science

#1 “breastfeeding” OR “exclusive breastfeeding”

#2 “pregnant” OR “antenatal” OR “prenatal” OR “postpartum” OR “postnatal” OR “new mother*”

#3 “Mobile Applications” OR “Telemedicine” OR “Text Messaging” OR “Cell Phone” OR “Smartphone” OR “mobile” OR “Portable Software Application” OR “Tele*” OR “mHealth” OR “eHealth” OR “e-health” OR “m-Health” OR “phone*” OR “app” OR “app” OR “app-based” OR “electronic” OR “web” OR “web-based” OR “Internet*” OR “digital*”)

#4 TS = clinical trial* OR TS= research design OR TS= comparative stud* OR TS= evaluation stud* OR TS= controlled trial* OR TS= follow-up stud* OR TS= prospective stud* OR TS= random* OR TS= (single blind*) OR TS= (double blind*)

#5 #1 AND #2 AND #3 AND #4

4. Cochrane Library (~medline) advanced search> search manager

#1 “exclusive breastfeeding”:ti,ab,kw

#2 breastfeeding [MeSH descriptor]

#3 pregnant OR antenatal OR prenatal OR postpartum OR postnatal OR new NEXT mother*:ti,ab,kw

#4 “Mobile Applications” [MeSH descriptor]

#5 “Telemedicine” [MeSH descriptor]

#6 “Text Messaging” [MeSH descriptor]

#7 “Cell Phone” [MeSH descriptor]

#8 “Smartphone” [MeSH descriptor]

#9 mobile OR portable NEXT software NEXT application* OR Tele* OR mHealth OR eHealth OR e-health OR m-Health OR phone* OR Text* OR “Short Message” OR SMS OR app OR apps OR “app-based” OR electronic OR Message* OR web OR “web-based” OR Internet* OR digital*:ti,ab,kw

#10 #1 OR #2 AND #3

#11 #4 OR #5 OR #6 OR #7 OR #8 OR #9

#12 #10 AND #11

5. CINAHL (via EBSCOhost)

#1 (MH "Breast Feeding+") OR TX ("exclusive breastfeeding")

#2 (MH "Pregnancy+") OR TX (pregnant OR prenatal OR antenatal)

#3 (MH "Postpartum Period+") OR TX (postpartum OR postnatal OR "new mother*")

#4 (MH "Telemedicine") OR (MH "Smartphone") OR (MH "Text Messaging") OR (MH "Mobile Applications") OR TX (mHealth OR eHealth OR e-health OR m-health OR phone* OR "mobile" OR "tele*" OR "Portable Software Application" OR Text* OR SMS OR app OR apps OR "app-based" OR electronic OR message* OR "web" OR "web-based" OR Internet* OR digital*)

#5 (MH "Randomised Controlled Trials") OR TX (randomised controlled trial OR controlled trial OR random* OR placebo* OR blind* OR trial*)

#6 #1 AND (#2 OR #3) AND #4 AND #5
