# Supplementary material for: Evaluating the Effectiveness of mHealth Application Interventions to Promote Breastfeeding Amongst Pregnant Women and Postpartum Mothers: A Global Systematic Review and Meta‐Analysis
Source: Matern Child Nutr. 2026 Aug 17;22(3):e70230. doi: 10.1111/mcn.70230 (PMC13480556; doi:10.1111/mcn.70230)
Supplement: Supplementary file 10 [file MCN-22-e70230-s010.docx]

**Supplementary Material 5**. Participant Demographics and Baseline Characteristics. (NR=Not Reported)

| **Study (Author, Year, Country)** | **Age (Mean ± SD)** | **Race/Ethnicity** | **Marital/**  **Partner Status** | **Education** | **Employment** | **Pregnancy Planned (%)** | **Mode of Delivery** | **Infant Sex** | **Prior BF (%)** | **Other Notes** |
| --- | --- | --- | --- | --- | --- | --- | --- | --- | --- | --- |
| UMICs and HIC studies (8 studies) | | | | | | | | | | |
| de Mello Sa, 2025, (US) | 33.4 ± 6.8 | Caucasian (56–75%), African-American (10–19%), Asian (5–19%), Other (6–10%); Hispanic (5–12%) | Mostly married (75–80%), some living with partner | High school 12–15%, college+ 85–88% | Employed ~50–60%, stay-at-home ~40–44% | Mostly planned (90–94%) | Vaginal (56–85%), C-section (15–44%) | Female ~40–44%, Male ~56–60% | 60–75% | Antenatal BSES-SF median ~52 |
| Lewkowitz, 2020 (US) | 22.7 ± 4.9 | 79–85% Black; 11% White; small % Hispanic, Asian, Other | 32–37% married/living with partner; ~28% partner but not cohabiting; ~32–39% single | 16–17% below high school; ~46–50% high school; ~25–31% some college; ≤6% college/graduate degree | 56–58% income <25k; 19% $25–50k; ~3–6% >50k; 90%+ smartphone ownership; ~45% unemployed/declined to answer | NR | NR | NR | NR | Planned infant feeding: ~49–54% breastfeeding only, 38–43% formula only, small % mixed/unsure |
| Uscher-Pines, 2019 (US) | 26.5 ± 5.22 | Non-Hispanic White 96%/ 97%; Hispanic 1%/ 2%  (% in intervention group/control group) | 53% married in intervention group, and 57% married in the control group | ≤High school 47%/ in the intervention group, and 43% in the control group | Income: <$15k 18%/ 14%; $15–25k 13%/ 10%; $25–40k 10%/ 16%; $40–55k 16%/ 14%; $55–80k 22%/ 15%; ≥$80k 11%/ 19%; Smartphone 97%/ 95%; Private insurance 48%/ 43%; Public 48%/ 51%; Uninsured 4%/ 4%  (% in intervention group/control group) | NR | Cesarean 47% in intervention group; and 40% in control group | Male 60% in intervention group; 47%  In the control group | Yes, 56% in intervention group; 57% in the control group | Primiparous 41% / 38%; GA ≥37 wks 86% / 86%; LBW <2500g 5% / 4%; Planned BF ≥3m 76% / 80%; ≥6m 70% / 74%; BF in 1st hr 79% / 87%; Exclusive BF during hospital stay 71% / 86% (p=0.01); Depression/anxiety 31% / 19%; Hypertension 9% / 2%; Smoked during pregnancy 19% / 14%  (% in intervention group/control group) |
| Baza, 2023 (US) | 24.5 ± 6.4 | 48% African-American, 43% White, 8% Hispanic, 3% Asian | 48% married | 60% secondary, 35% university | 45% employed; 48% unemployed | 55% planned pregnancy | Cesarean 25% | Male 44% | NR | Mean GA 38.7w; Mean BW 3433g; Median time to BF initiation 0.5h |
| Uscher-Pines, 2025 (US) | NR | American Indian or Alaska Native (3.7%/2.4%), Asian (5.1%/5.0%), Black or African American (31.5%/32.4%), Latinx (61.7%/57.8%), Middle East or North African (1.3%/0.7%), Native Hawaiian or Pacific Islander (0.9%/0.6%), White (53.3%/49.6%), Multiple ethnicities (45%/43.2%), Other race (16.4%/18.4%)*  Black including black latinx (31.5%/32.4%), Latinx (non-black) (35.8%/35.1%), Non-black or Non-latinx (32.7%/32.4%)**  (% in intervention group/control group) | Married 61.7% in intervention group, and 57.8% in the control group | High school or less (13.0%/12.8%), Some college (28.2%/30.0%), 4 year college (32.8%/28.8%), Graduate degree (26.1%/28.5%)  (% in intervention group/control group) | NR | NR | Cesarean 30.4%/32.1%  (% in intervention group/control group) | Male 52.9%/51.2%, Female 45.6%/47.6%  (% in intervention group/control group) | NR | Smoked during pregnancy 2.4%/3.5%; Living at Large city (33.5%/34.2%), Suburb (35.6%/35.9%), small city (25.7%/24.9%), Rural area (5.2%/5.0%)  (% in intervention group/control group) |
| Yikar, 2024  (Turkey) | NR | NR | NR | Primary/secondary majority, small proportion bachelor+ | Mostly non-working 88–96% | Planned 88–96% | NR | NR | NR | / |
| Acar, 2024 (Turkey) | 27.7 ± 4.5 | NR | NR | High school or university: 78% / 84%  (% in intervention group/control group) | Employed 36% / 43%  (% in intervention group/control group) | NR | NR | NR | NR | / |
| Vila-Candel, 2024 (Spain) | 33.2 ± 4.4 | Spanish 28%/ 72%, Foreign 8%/ 92%  (% in intervention group/control group) | Married 22% / 65%  (% in intervention group/control group) | University 33% / 55%, Primary/Secondary 15% / 85%  (% in intervention group/control group) | Employed 25% / 75%, Unemployed/student 22% / 78%  (% in intervention group/control group) | NR | C-section 19%/ 26%, Eutocic 27%/ 73%, Instrumental 21%/ 79%  (% in intervention group/control group) | Male 21%/ 79%, Female 27%/ 73%  (% in intervention group/control group) | Yes, 21%/ 79%  (% in intervention group/control group) | Primiparous 28% / 72%, Multiparous 16% / 84%  (% in intervention group/control group) |
| LMICs studies (5 studies) | | | | | | | | | | |
| Musiimenta, 2022 (Uganda) | 27 (SD NR) | NR | Married/cohabiting 83–95%, small % separated/single | Mostly primary education (93–98%); few with no education, Limited literacy (68–78% unable to read English; ~95% could read Runyankole) | All had no regular income; food insecurity common (73–75%) | NR | NR | NR | NR | HIV+ (8–15%); ANC visit at 3–4 months; distance to facility 8–20 km; spouse decision-making 66–79%, Rural, low-income setting |
| Sosanya, 2025 (Nigeria) | NR | 100% Nigerian | Married 88–94%; Single/separated/cohabiting 6–12% | Secondary 72%; Primary 16–17%; None 7–10%; Tertiary 3% | Personal income mostly ≤USD12 (56.7% in control group, 54.6% in intervention group); Household income ≤USD12 (34.0% in control group, 41.2% in intervention group) | NR | NR | Male 52–60%; | NR | Child age 0–23 months |
| Seyyedi, 2021 (Iran) | 26 (SD NR) | NR | NR | Middle school 33–38%, Diploma 30–40%, Academic 23–38% | Employed 8–13%, Home-employed 8–23%, Housewife 70–80% | NR | NR | NR | NR | Income: Cost>Income 43–55%, Cost=Income 38%, Cost<Income 8–8%; Residency City 5–8%, City margin 13–33%, Village 60–83% |
| Seddighi, 2022 (Iran) | 29.8 ± 5.1 | NR | NR | Primary 1–5%, Elementary 4–9%, Secondary 5–7%, Diploma 41% / 42%, University 47% / 40%  (% in intervention group/control group) | Housewife 69% / 82%, Other employment 28–31%  (% in intervention group/control group) | NR | Vaginal 29% / 25%, Cesarean 71% / 75%  (% in intervention group/control group) | NR | NR | Family income 1–2+ million 28–44% / 30–37%  (% in intervention group/control group) |
| Abadi, 2024 (Iran) | 28 | NR | NR | Diploma 45% / 35%, Bachelor 45% / 40%, Master+ 10% / 25%  (% in intervention group/control group) | Employed 55% / 70%, Housewife 45% / 30%  (% in intervention group/control group) | NR | NR | Female 55%/ 5%, Male 80%/ 20%  (% in intervention group/control group) | NR | Residency: City 40% / 55%, Village 60% / 45%  (% in intervention group/control group) |

*Race and ethnicity that are not mutually exclusive.

**Race and ethnicity that are mutually exclusive.
